# Telerehabilitation use and experiences in occupational and physical therapy through the early stages of the COVID-19 pandemic

**DOI:** 10.1371/journal.pone.0291605

**Published:** 2023-11-08

**Authors:** Golda Nguyen, Katelyn King, Leia Stirling

**Affiliations:** 1 Department of Aeronautics and Astronautics, Massachusetts Institute of Technology, Cambridge, Massachusetts, United States of America; 2 Department of Biomedical Engineering, Department of Robotics, University of Michigan, Ann Arbor, Michigan, United States of America; 3 Department of Industrial and Operations Engineering, Department of Robotics, University of Michigan, Ann Arbor, Michigan, United States of America; Transilvania University of Brasov: Universitatea Transilvania din Brasov, ROMANIA

## Abstract

Telehealth has helped to increase access to rehabilitative services such as occupational and physical therapy. The early COVID-19 pandemic amplified the need for remote access to care, and the rapid implementation of telehealth systems provided a unique opportunity to learn from clinicians’ experiences adopting telehealth for telerehabilitation applications. To understand these experiences, a self-administered online survey was conducted to capture perspectives on ease of telerehabilitation use and adoption from occupational and physical therapists. The survey captured retrospective views on telerehabilitation use pre-pandemic as well as real-time perspectives on telerehabilitation during the early stages of the pandemic (July to August 2020). The survey gathered information on clinician demographics (N = 109), clinicians’ experiences with adopting or utilizing telerehabilitation systems, and their perceptions on remotely performing cognitive, emotional, and physical assessments via video-conferencing (a common mode of telehealth). Responses demonstrated a modest increase in telerehabilitation as a care setting (rate increase from 3.4% to 19.3%), and telerehabilitation was more generally tried during the early stages of the pandemic (41 clinicians explicitly reported telerehabilitation use). However, technology access and acceptance remained low, with 38 clinicians (35%) expressing concerns that technology was ineffective or impractical, unavailable, not covered by insurance, or not desired by their patients. Video-conferencing technology was perceived as generally ill-equipped to support clinicians in performing remote assessment tasks. Physical assessment tasks were considered particularly difficult, with 55% of clinicians rating their ability to perform these tasks in the range of moderately difficult to unable to perform. To address these difficulties and better augment clinical care, clinicians require more robust assessment methods that may combine video, mobile, and wearable technologies that would be accessible to a patient at home. When designing future telerehabilitation tools, information captured through these modes must be task-relevant, standardized, and understandable to a remote clinician.

## Introduction

Telerehabilitation is a form of telehealth that provides an alternative to in-person rehabilitative care by connecting practitioners and patients remotely through telecommunications systems [1]. Recent trends indicate that clinicians underutilize technology in rehabilitative applications [2]; however, the COVID-19 pandemic initially altered this pattern by disrupting in-person care and shifting some aspects of care delivery to a virtual setting. This transition presented an opportunity to expand the potential benefits of telehealth by learning from successes and challenges of telerehabilitation implementation and adoption during a period when remote care was necessary.

The efficacy of care via telerehabilitation has varied with the condition being treated but has been demonstrated for a variety of pathologies [3,4], including musculoskeletal [5], neurologic [6], and cardiac-related [7] conditions. Synchronous telerehabilitation programs have been utilized by individuals with musculoskeletal conditions to improve physical function and reduce pain [5,8], and by adults with traumatic brain injuries to improve mood, communication, and sleep quality [9]. Shigekawa et al. [3] and Corey [10] found that clinicians generally expressed positive views of the potential of telerehabilitation to provide more cost-effective, timely, and accessible care to a broad patient population. In a survey (n = 310) of Florida-based physical therapists (PTs), respondents largely agreed that telehealth could substantially reduce healthcare costs for patients (81%) and make delivering quality care more efficient (89%) [1].

However, barriers to telehealth implementation including user apprehension, inexperience with technology, technical difficulties, confidentiality concerns, and regulatory limitations [10,11] have resulted in low utilization of telerehabilitation in practice [1,11,12]. A survey of California-based occupational therapists (OTs) found that participants with telehealth experience identified client and practitioner apprehension and technology difficulties and challenges as the greatest barriers to telehealth implementation [10]. Surveys of other OT and PT populations reinforce these concerns: 46% of Houston-based OTs (n = 51) felt unequipped to conduct a productive consultation over telehealth [11], while 90% of Florida-based PTs (n = 310) reported having little or very little experience with telehealth technology [1]. Technical difficulties have also posed challenges, with one survey finding technical difficulties affecting up to 47% of respondents [1]. In their survey of Florida-based OTs, Corey [10] reported that OTs reserved telehealth consultations for making-up sessions, avoiding illness, or reducing travel time, rather than making telehealth regularly available.

While the onset of the COVID-19 pandemic was met with continuing concerns from clinicians, there were also promising new trends in telehealth [13]. During the early stages of the pandemic, some facilities increased the proportion of physical therapy sessions being conducted via telehealth and received positive responses from patients on satisfaction with remote care. For example, three clinics belonging to the University of California San Francisco Medical Center (UCSFMC) [14] reported increasing telehealth use to 85% of sessions provided with high satisfaction on telerehabilitation sessions. Bay State Physical Therapy (based in the New England area) provided 23,000 telerehabilitation sessions in 14 weeks from March to June 2020 and reported that patients valued the convenience and safety of telerehabilitation with ratings as high as 95% satisfaction for care received via video-conferencing (a common mode of telehealth used for telerehabilitation) [15]. However, with social-distancing restrictions, it remained unclear whether practitioners perceived the transition to telehealth as a permanent shift in their practice or an adequate solution for the interim. These data prior to and during the pandemic support a need to understand and delineate the challenges associated with telerehabilitation systems and tools. The objective of this work was to capture insights from a broader geographical community of OTs and PTs on telerehabilitation adoption and implementation during the early pandemic and to examine perceptions on performing common clinical assessment actions (cognitive, emotional, physical) via video-conferencing, which is a common mode of telehealth.

## Methods

A self-administered survey was developed to capture perspectives from OTs and PTs on care delivery and telerehabilitation experiences before and during the early stages of the COVID-19 pandemic. Survey methods were used to allow ease of participation during the early pandemic and to seek a wide sample of clinician experiences that would not be reachable through in-person experimental methods. A survey was used over other qualitative methods to consistently capture perceptions of clinicians in a brief window of time with flexibility for respondents to participate on their own schedule. The survey was disseminated to practicing OTs, PTs, and relevant academic connections through email, online forums focused on OT or PT engagement/professional networks (World Federation of OTs, OT International Online Network, Reddit Physical Therapy, Reddit Occupational Therapy), and social media outlets (Twitter, Facebook). Recruitment emails and posts through all mediums mentioned were only conducted once per medium. The target audience for the survey was practicing OTs and PTs, but no specific targeting was conducted on where or how clinicians practice to obtain a broad range of experiences and geographic spread in the sample. The survey was deemed exempt from IRB oversight by the University of Michigan Health Sciences and Behavioral Sciences Institutional Review Board. Consent was not obtained, but participants were provided with information about the voluntary study on the opening page of the online survey and could continue with the survey after reading this initial information. Responses were accepted from July 15, 2020, to August 20, 2020. Data were anonymized at data collection, with no identifying information collected.

### Survey design

The survey was developed in Qualtrics Survey Software (Provo, UT) and consists of 42 questions grouped into three major sections: (1) demographics, (2) retrospective insights on clinical experiences before the pandemic (“Pre-COVID”), and (3) clinical experiences during the early stages of the pandemic (“During COVID”). Responses to all questions were optional, so participants could elect to not respond to any question. Location data were collected through IP address upon survey submission through Qualtrics, but no personal data were collected. The full survey can be found in supplementary material S1 File.

The demographic section asks 7 questions about the respondent’s age (Q1), sex (Q2), healthcare professional area (Q3), how long they have been practicing (Q4), the types of cases they see (Q5), the age range of patients they see (Q6), and the geographical area in which they live (Q7). Q1-Q4 and Q7 were asked as single-choice response questions. For Q5 and Q6, respondents could select from multiple-choice responses as matches their situation. The “Pre-COVID” and “During COVID” sections included questions about care settings (Q8/Q19), the weekly average number of patients (Q10/Q20), average length of a clinical appointment (Q11/Q21), types of interaction with patients (Q12/Q13/Q24/Q25), technology use for patient assessment (Q14/Q15/Q26/Q27/Q33), and reasons for using or not using telehealth (Q9/Q28/Q47).

In addition to the previous list, respondents were explicitly asked about changes to their experiences when remote work or social distancing policies were enacted. These questions specifically asked about caseload changes for face-to-face versus virtual appointments (Q16/Q17/Q18/Q30), changes to types of activities prescribed (Q31/Q32), changes to health insurance policies (Q22/Q23), and perceptions on information gained or lost during virtual patient interactions (Q35/Q36/Q37/Q38/). If a respondent indicated that they used remote or virtual practice during the COVID-19 pandemic, they were also asked a series of 20 items on a 7-point Likert scale (Q39/Q48) to rate their ability to perform cognitive, emotional, and physical assessment actions via video-conferencing. The items presented were based on physical, emotional, and cognitive features that OTs commonly assess as observed by Stirling and MacLean [16]. The work of Stirling and MacLean [16] was incorporated in this survey to provide content validity for mapping clinical actions to survey measures on perceived capabilities and limitations of remote care. Pilot testing was performed on survey questions with a non-clinical population to evaluate wording clarity and survey logical flow.

### Survey analyses

Survey responses were included if the respondent answered questions beyond the demographic section (n = 109). Each question was analyzed based on the number of clinicians who elected to respond. Specific analyses were conducted to examine changes to caseload, distribution of and changes to care settings, and perceptions on remote assessment abilities via video-conferencing.

In the survey, clinicians identified the care settings in which they supported patients Pre-COVID and During COVID. No explicit definition of care setting was provided; instead, clinicians were asked to select among multiple care setting options based on documented specialties that are available: inpatient, outpatient, in-home healthcare, nursing home, school system, telehealth, other. Thus, responses represent clinicians’ personal interpretations of what constitutes their care setting. Comparing responses from Pre-COVID and During COVID periods, the difference in care setting distribution was evaluated using a 2×6 contingency table with a chi^2^ test (the category of “Other” was removed for this analysis). Post-hoc evaluation was examined using rate ratios and difference in rates.

The Likert scale items were grouped into three sub-categories on perceived ability to perform cognitive, emotional, and physical assessments. Cognitive items prompted respondents to provide ratings on their ability to assess patients’ awareness, responsiveness, comprehension of directions or cues, and ability to follow directions and communicate with their clinician. Emotional items examined clinicians’ abilities to interpret patient emotion (frustration, fatigue, pain), and physical items examined clinicians’ abilities to observe patient motion (manual dexterity, compensatory mechanisms, gross motor function, active range of motion), coordination (balance, motion fluidity/smoothness, coordination patterns within and across limbs), and physical condition (spasticity, sensation, muscle tone, strength, muscle activation patterns, posture). The number of individuals that responded to each item was reported as a percentage of the total number responses for that specific item. Additionally, overall perceptions were calculated for the three major categories (cognitive, emotional, physical) by aggregating the responses from all items within each category.

## Results

The survey received 135 total responses. Participants were included in analysis (n = 109) if they submitted responses beyond the demographic section. Inclusion into the analysis sample does not necessarily indicate whether the respondent explicitly denoted using or not using telehealth. A detailed examination of respondents’ experiences performing common OT/PT assessment tasks via video-conferencing is also presented (n = 36). It is important to note that sample sizes are explicitly displayed and may differ by topic or question posed as respondents were not required to answer all questions. Therefore, the differences in sample sizes for responses may be due to participants skipping a question or not being prompted to answer a question based on survey logic (e.g. indicating whether they did or did not utilize telehealth).

### Demographics

Demographic information (summarized in Table 1) indicated a range of respondents but demonstrated strong skews in clinician gender and age of patients treated. The respondents were predominantly female (72%) and between 18–34 years old (82%). The survey group also skewed toward newer clinicians, with 64% having <5 years of experience. Clinicians were mostly located in suburban (53%) or urban (34%) communities, and nearly all respondents (95%) were based in the United States (US) with only 5 respondents based outside of the US (5%). Within the US, respondents represented 38 states, with geographical spread across all major regions of the United States as defined by the US Census Bureau Regions and Divisions [17]. More specifically, 22 respondents were based in the West region, 33 respondents in the Midwest region, 31 respondents in the South region, and 18 respondents in the Northeast region.

**Table 1:** Demographic information of survey respondents.

Profession *(n = 109)*	Count (%)
Occupational Therapist (OT)	49 (45)
Physical Therapist (PT)	58 (53)
Student Therapist	2 (2)
**Age** *(n = 109)*	**Count (%)**
18–34 years old	89 (82)
35–64 years old	20 (18)
**Gender** *(n = 109)*	**Count (%)**
Female	79 (72)
Male	29 (27)
Non-binary	1 (1)
**Experience** *(n = 109)*	**Count (%)**
*<*1 year	17 (16)
1–5 years	52 (48)
5–10 years	29 (27)
*>*10 years	11 (10)
**Geographical Location** *(n = 109)*	**Count (%)**
Rural	14 (13)
Suburban	58 (53)
Urban	37 (34)

Demographic information on survey respondents was captured in the areas of profession, age, gender, experience, and geographical location.

Questions on pathologies and patient demographics allowed clinicians to select multiple responses. The five most common pathologies supported were orthopaedics (50%), physical rehabilitation (50%), stroke rehabilitation (37%), brain injury or neurology (37%), and pediatrics (28%). The demographics of the patients cared for included 14% working with infants/toddlers (ages 0–3 years), 27% working with children (ages 4–10), 34% working with adolescents (ages 11–18), 61% working with adults (ages 18–65), and 73% working with older adults (over 65).

### Telehealth use before and during the early stages of the COVID-19 pandemic

Clinicians surveyed indicated an increase in telehealth use as a care setting and a higher likelihood of using telehealth during the early stages of the pandemic. Pre-COVID, only 5 out of 107 clinicians (5%) reported telehealth as a care setting (Table 2). During the early stages of the COVID-19 pandemic, 22 out of 85 respondents (26%) noted telehealth as a care setting (Table 2). In response to a specific question about whether clinicians used remote or virtual practice (telehealth, telemedicine, etc.) during the pandemic, 41 out of 82 clinicians (50%) reported using telehealth at some point during the early stages of the pandemic (Table 3). Another 41 clinicians (50%) responded to this specific question as having not used telehealth at the time of survey completion. These responses were distinct from noting telehealth as a care setting. The difference in responses for telehealth use versus telehealth as a care setting suggests that clinicians perceived trying telehealth as distinct from adopting and sustaining this mode in their practice.

**Table 2:** Clinical care settings and geographical location prior to and during the early stages of the COVID-19 pandemic.

Period (Geography)	Inpatient	Outpatient	In-home	Nursing Home	School System	Telehealth	Other
**Pre-COVID (Total)** *(n = 107)*	**32**	**61**	**18**	**18**	**14**	**5**	**4**
Pre-COVID (Rural) *(n = 14)*	4	7	2	5	1	1	0
Pre-COVID (Suburban) *(n = 56)*	12	36	8	7	10	2	1
Pre-COVID (Urban) *(n = 37)*	16	18	8	6	3	2	3
**During-COVID (Total)** *(n = 85)*	**21**	**41**	**13**	**11**	**6**	**22**	**6**
During-COVID (Rural) *(n = 10)*	2	4	1	4	0	2	1
During-COVID (Suburban) *(n = 47)*	9	25	5	5	6	12	2
During-COVID (Urban) *(n = 28)*	10	12	7	2	0	8	3

Respondents reported clinician care settings and geographical location prior to and during the early stages of the COVID-19 pandemic, and respondents could select multiple care settings if relevant to them.

**Table 3:** Clinician responses to telehealth use during the early pandemic as well as reasons for not using telehealth during the early pandemic.

**Explicit Telehealth Use During**	**Number of**
**COVID-19** *(n = 82)*	**respondents** (%)
Yes	41 (50)
No	41 (50)
**Reason for not using telehealth**	**Number of**
**during the COVID-19 pandemic** *(n = 38)*	**respondents** (%)
Technology is not effective or practical	16 (42)
Technology is not available	11 (29)
Lack of insurance coverage	11 (29)
Other	12 (32)
Employer restrictions	5 (13)
Lack of patient interest or technology	3 (8)
Prefer in-person care for geriatric patients	2 (5)
Other, unspecified	2 (5)

Clinicians (n = 82) responded to whether they had or had not used telehealth during the early stages of the COVID-19 pandemic. For clinicians who reported not using telehealth during COVID-19, 38 out of 41 respondents provided motivations for not using telehealth. Respondents could select from multiple reasons if relevant to them or provide "Other" motivations in an open-response section, as specified in the lower portion of the table.

Prior to the pandemic, telehealth was not a predominant care setting (5%). Respondents largely practiced in outpatient care (57%), and then were spread across inpatient care (30%), in-home care (17%), nursing home facilities (17%), school systems (14%), and other care settings (4%) (Table 2). There was a difference between care settings due to the pandemic (chi^2^(5) = 133.2, p<0.001). During COVID, clinicians were 5.7 times more likely to use telehealth (rate increase from 3.4% to 19.3%) as compared to Pre-COVID. Rate decreases were observed during the pandemic for Inpatient (3.2%), Outpatient (5.3%), In-home (0.8%), Nursing Homes (2.5%), and School Systems (4.2%).

Despite increased telehealth use, the majority of respondents (80%, 71 of 89) still reported seeing patients face-to-face during the early stages of the pandemic. Of the 71 clinicians who continued seeing patients face-to-face, 37 (53%) reported seeing fewer patients face-to-face, 29 (41%) saw the same number, and 4 (6%) saw more patients. Of the clinicians who reported using telehealth during the early stages of COVID (n = 41), a majority continued interacting with patients face-to-face (73%, 30 of 41). Even among clinicians who regarded telehealth as a care setting during the early stages of COVID, 73% (16 of 22) continued treating patients face-to-face.

Another aspect of care delivery that clinicians maintained during the pandemic were their methods of assessing patients (Table 4). Pre-COVID and During COVID, clinicians reported similar levels of use for visual observation (98% Pre-COVID to 95% During COVID) and patient self-reports (85% Pre-COVID and During COVID). Clinicians’ use of standardized tests increased during the early stages of COVID from 78% to 90% (Table 4), although it is unclear in which care settings these increases occurred. However, one clinician commented that “[s]tandardized tests [are] made unstandardized through virtual application.” Another echoed this concern, reporting that they are “[s]till working on… finding a standardize [sic] assessment that could work remotely.”

**Table 4:** Reported methods of assessing patient state or progress during rehabilitation.

	Number of respondents (%) *(n = 88) (n = 82)*	
Assessment Mode	Pre-COVID	During COVID
Visual observation	86 (98)	78 (95)
Standardized tests	64 (78)	79 (90)
Patient self-reports	75 (85)	70 (85)
Other	7 (8)	11 (13)
Teacher/caregiver reports	7 (8)	10 (12)
Other, unspecified	0 (0)	1 (1)

Clinicians reported methods of assessing patient state or progress over the course of rehabilitation before and during the pandemic (respondents could select multiple methods if relevant to them). Here *n* indicates the number of responses included in each time period.

Clinicians also expressed concerns that led them to not use telehealth during the early pandemic at all. Of the 41 clinicians who did not use telehealth during COVID (Table 3), 38 provided rationale including that telehealth technology is not effective or practical (16, 42%), or technology or insurance coverage was not available (11, 29%). Two respondents (5%) explicitly noted lack of patient interest, while 5 (13%) cited their employer’s lack of support for telehealth.

### Perceptions of assessment ability via video-conferencing

**Fig 1:**
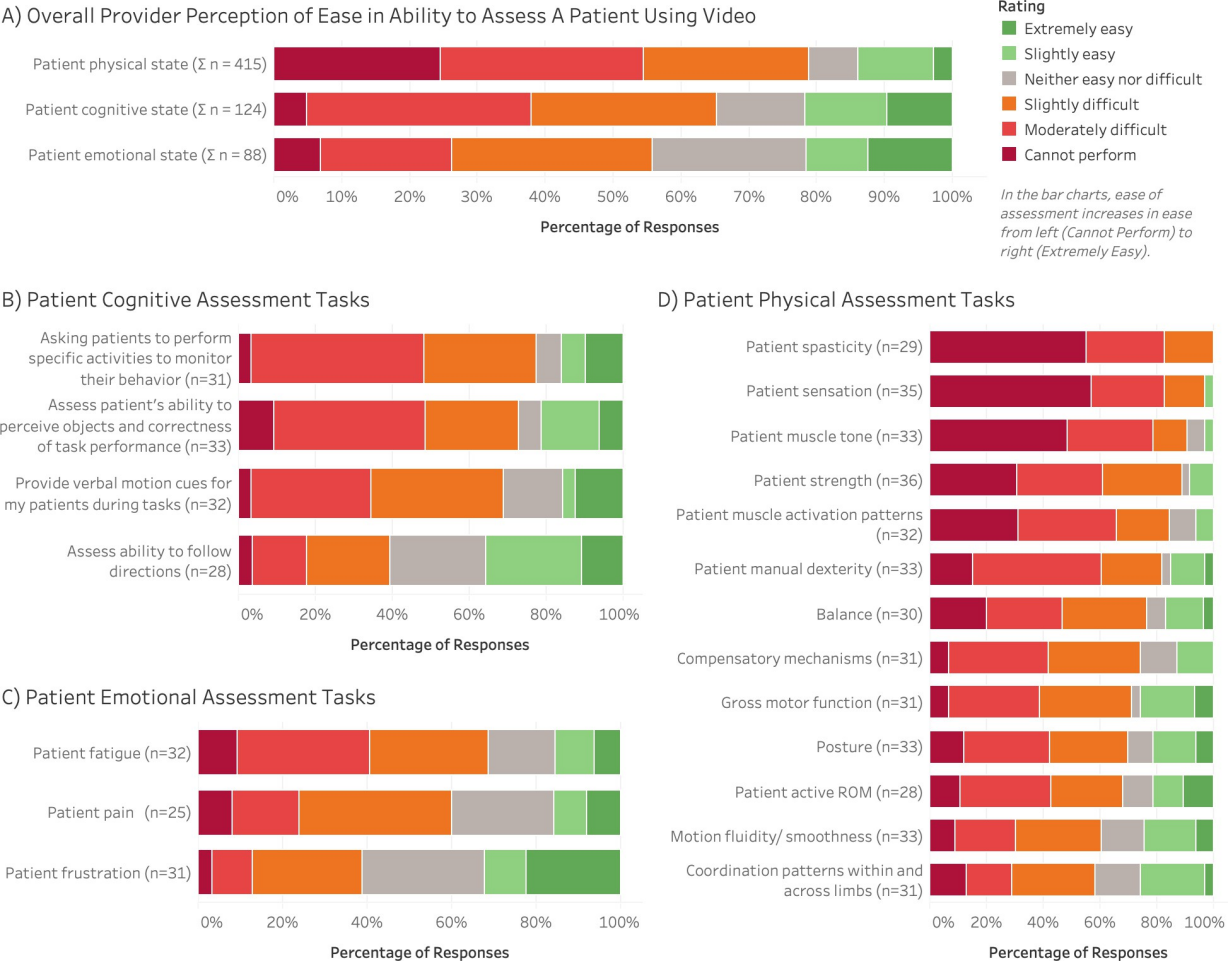
Perceptions of clinician’s ability to perform cognitive, emotional, and physical assessments through video-conferencing. Respondents who reported using telehealth during the early stages of the pandemic were asked to rate their ability to perform specific task assessments over video-conferencing on a 7-point Likert scale (Extremely easy, Moderately easy*, Slightly easy, Neither easy nor difficult, Slightly difficult, Moderately difficult, Cannot perform). Scale items (tasks) were categorized into cognitive, emotional, and physical assessments for analysis. (a) A composite summary of ratings aggregated from each of the three categories. (b) Ratings for cognitive assessments tasks. (c) Ratings for emotional assessment tasks. (d) Ratings for physical assessment tasks. The number of responses *n* is included for each task. Not all respondents provided ratings for all tasks. *No respondents selected “Moderately easy” for any tasks; therefore, it is not shown in the Rating legend or bar charts.

Clinicians who used remote or virtual practice during the early stages of the pandemic (Table 3) were asked to provide ratings on a Likert scale about their ability to perform specific tasks for cognitive, emotional, and physical rehabilitation assessments through the use of video-conferencing (Fig 1). When aggregating all responses to all tasks (Fig 1A), the majority of responses indicated that physical assessment tasks (79%), cognitive assessment tasks (65%), and emotional assessment tasks (56%) were slightly difficult, moderately difficult, or not performable via video-conferencing. While the survey provided a 7-point Likert scale for respondents to rate tasks on (Extremely easy, Moderately easy, Slightly easy, Neither easy nor difficult, Slightly difficult, Moderately difficult, Cannot perform), no participants responded to a task as “Moderately easy”, and therefore this rating is not shown in Fig 1.

Respondents reported the most difficulty with physical assessment tasks. When pooling all physical assessment tasks (Fig 1A), 25% of clinicians reported that they could not perform physical assessment tasks via videoconferencing compared to the “Cannot perform” responses of 5% and 7% for overall cognitive and emotional assessment tasks, respectively. Respondents also reported the greatest difficulty with physical assessment tasks that would normally involve a tactile component if conducted in-person. More specifically, the proportion of clinicians that responded with a rating of at least slightly difficult was highest for assessing patient spasticity (100%), sensation (97%), and muscle tone (90%).

For cognitive assessment tasks (Fig 1B) and emotional assessment tasks (Fig 1C) that would be performed via videoconferencing, respondents predominantly reported tasks to be “Slightly difficult”, “Moderately difficult”, or “Cannot perform”. The tasks with higher ratings of difficulty included asking patients to perform specific activities to monitor behavior, assessing patient’s ability to perceive objects and correctness of task performance, providing verbal motion cues, assessing patient fatigue, and assessing patient pain. Conversely, participants rated the tasks of assessing ability to follow directions and assessing patient frustration with greater ratings of ease.

## Discussion

This survey captured insights on the increased use of telehealth as a care setting for telerehabilitation during the early stages of the COVID-19 pandemic as well as insights on current challenges in OT/PT care delivery with video-conferencing. Clinicians’ perspectives highlighted opportunities to augment clinical care and gaps in the accessibility and desirability of telehealth as a mode of care delivery for rehabilitation. Their experiences with video-conferencing also highlight limitations to effectively delivering care through this mode alone, particularly when seeking to perform assessments of patient physical state. Perceptions observed in the survey spanned Likert responses from perceived ability to perform tasks with ease to being unable to perform the task with video-conferencing alone. These insights reinforce the need for expanding assessment capabilities (with additional modalities or technologies) and further developing accompanying standards to consistently capture and convey key information on remote patient state to clinicians. While telehealth systems have delivered great benefit to patients and providers by lowering barriers to accessing care, the aforementioned needs must be addressed to sustain telehealth use and adoption. To achieve effective tools and sustained adoption, telehealth designers should consider multi-faceted approaches that combine in-person care with remote video, mobile, and wearable technologies to jointly address clinician and patient needs.

### Telehealth use and desirability

It is evident that telehealth use increased during the COVID-19 pandemic, but a distinction exists between use and sustained adoption. This distinction seemed to be influenced by variation in individual definitions of telehealth or virtual practice. While 41 clinicians explicitly reported using virtual practice (Table 3), only 22 clinicians considered telehealth as a care setting during the early stages of the pandemic (Table 2), possibly indicating different perceptions of what constitutes as telehealth as well as indicating gaps in initial implementation versus sustained use of telehealth (as a care setting). These variations may also be influenced by organizational and policy barriers to implementation and acceptance. Compared to the 85% implementation that was observed in the UCSFMC Outpatient Physical Therapy Family Practice [14], this survey revealed more moderate gains in telehealth usage with only 50% of respondents using it during the early stages of the pandemic (Table 3). The lower rate of telehealth use observed in this study may be partly attributed to lack of institutional support. Respondents who reported unique reasons for not using telehealth (Table 3) commonly cited employer restrictions, including that their employer “never made [telehealth] available”, did not “focus on” or “support” implementation, or took “several months to roll out” telehealth systems. Hospitals may be wary of investing in telehealth systems due to uncertainty in patient interest and coverage (which may be driven by insurance reimbursement policy). As has been seen before [18] and during the pandemic [19], organizational influences (such as company attitudes, financial limitations, and policy factors) continue to present barriers to telehealth adoption and provide important context on constraints to improving and implementing future systems.

Additionally, telehealth as a care setting for rehabilitation did not fully replace in-person care. The majority of clinicians surveyed were still interacting with their patients face-to-face during the pandemic, particularly in outpatient and inpatient care (Table 2). One clinician noted that even though their clinic “offered Zoom appointments… everyone has just been coming in person” (Table 3). These results support how even amid the social-distancing restrictions of a global pandemic, in-person interaction was still the preferred method of delivery for rehabilitative therapy services.

### Technology limitations and opportunities

Beyond organizational and policy factors, this survey highlighted how the technology medium for conducting telerehabilitation can present challenges to performing fundamental tasks to assess patients. This study specifically examined clinicians’ perceptions of performing tasks via video-conferencing as the predominant telehealth modality implemented prior to and during the early stages of the pandemic. Respondents with telehealth experience overwhelmingly regarded tasks performed with video-conferencing as difficult (Fig 1). Activities with the highest proportion of difficulty ratings, such as assessing patient spasticity, sensation, and muscle tone (Fig 1D), are tasks that would typically require palpation or tactile methods when performed in-person. Therefore, clinicians lacked a major stream of information on patient state that was not sufficiently being augmented through video-conferencing. Even among physical tasks that would typically be assessed via visual observation if performed in-person (manual dexterity, balance, compensatory mechanisms, gross motor function, posture, range of motion, fluidity, coordination patterns), the majority of clinicians still considered these tasks to be at least slightly difficult to perform by video. This perceived difficulty could arise from the limitations in the video viewpoint, which would affect depth cues, observability of task-specific body segments, and ability to dynamically change perspective. Clinicians generally associated greater ease with cognitive (Fig 1B) and emotional (Fig 1C) assessments compared to physical assessment but still predominantly perceived them as difficult through video-conferencing. For cognitive assessment tasks, respondents expressed the greatest difficulty with monitoring patient behavior to assess cognitive understanding of the task. These tasks included the clinician being able to observe the patient’s directional attention to assess if they are perceiving the necessary information, as well as the ability to perform the task steps following the provided instructions. In these scenarios, limited viewpoints from the video may also affect these interpretations. For emotional assessment tasks, the majority of respondents expressed the greatest difficulty with assessing fatigue and pain, even though these assessments do not typically involve gathering tactile information when performed in-person. The range of perceptions observed for these dimensions of physical, cognitive, and emotional assessments emphasizes the complexity of interactions that influence telerehabiltiation usability and acceptance. Differences in clinicians’ personal experiences, including different tools, training methods, and patient interactions, may drive the observed variability in perceptions.

Overall, the predominant perceptions of difficulty in this study support existing literature [11,20] indicating that video-conferencing alone is insufficient for virtual care. The comparison of response distributions for each assessment task (Fig 1) is intended to be a roadmap for technology developers to understand where video tools struggle the most and where other modalities may help augment. Additional technologies, such as mobile devices, wearable technologies, and personalized robotics [16,21,22], have demonstrated potential to expand the set of information captured on a remote patient and provide more detailed insight for a clinician to make an informed assessment. Of course, for such technology systems to be effective, they require robust algorithms to support case-specific decision making and metrics that can be personalized to a patient’s individual needs. Effective telerehabilitation systems must also establish replicable methods of care delivery to address the lack of standards for assessing patients virtually. Clinicians in this study expressed concerns that many common assessments did not transfer well into a virtual environment. Similarly, in an interview of Floridian OTs Pre-COVID, multiple providers noted that “there is no regulation right now,” leaving clinicians to devise their own methods of delivering virtual care [10]. The lack of standardized assessments for virtual settings presents an opportunity for new technologies to either recreate standard measures or develop and validate new, intuitive methods. Expanding efforts to validate and standardize telerehabilitation tools should be a primary focus for telehealth system development going forward to sustain the use and benefits of telehealth beyond the pandemic.

### Limitations and future work

At the time that the survey was administered, the duration of the COVID-19 pandemic could not be predicted. Therefore, the results of this survey are limited in scope as they only capture the early stages of the pandemic. Future work should evaluate the evolution of telehealth adoption in OT and PT over the course of the pandemic and contextualize the current outlook for telerehabilitation use and acceptability. Additionally, the results of this survey revealed a discrepancy in the number of respondents who reported trying telehealth and those who considered telehealth as a care setting in which they consistently support patients. This nuance highlights variations in how respondents may have individually defined or perceived what telehealth may involve. The discrepancies in response may be mitigated by providing definitions of terminology to standardize possible interpretation.

An additional limitation exists with the Likert scale used to capture perceptions on performing cognitive, emotional, and physical assessment tasks. The scale items do not comprise an exhaustive list of potential items that OTs and PTs may assess with patients. In particular, the cognitive and emotional domains may be expanded on with constructs from scales such as the Canadian Occupational Performance Measure (COPM) [23], the Mini-Mental Status Examination (MMSE) [24], the Montreal Cognitive Assessment (MoCA) [25], and the Loewenstein Occupational Therapy Cognitive Assessment (LOTCA) Battery [26]. Manee et al. [27] found COPM, MMSE, and MoCA to be most predominantly used amongst a global survey of occupational therapists, but the availability of and clinicians’ familiarity with certain scales should be considered when choosing a scale. These highlighted constructs are also contextualized for different impairments, so future surveys and validation studies may consider selecting certain constructs to evaluate the focus on a specific category of illness or injuries. Incorporating additional constructs on cognition, mental health, and psychomotor ability may provide further insights for assessments of clinical usability of telerehabilitation. Furthermore, the present study only considered perceptions of telehealth use as a care setting, but future studies should experimentally evaluate telerehabilitation validity through inter-rater and intra-rater assessment of patient outcomes by clinicians when using video assessments.

Additional limitations existed with the geographical spread of respondents. While respondents represented OTs and PTs from a range of geographic areas (rural, suburban, and urban as well as across all geographic regions of the US and some international locations), the survey sample was predominantly from suburban and urban communities. Therefore, insights on unique challenges for clinicians serving rural patients or characterizations of specific geographic areas were not generalizable.

As a voluntary survey, this study was also limited by potential selection bias and the number of respondents. It was not feasible to calculate response rate because the total possible sample of people reached through recruitment could not be captured, preventing the ability to assess external validity through response rates or bias. Response bias may have also been present across each individual question, as respondents were allowed to skip questions. Adding questions within the survey, such as additional constructs or alternate phrasing for evaluating internal consistency, should be balanced against response fatigue. For the current survey, the questions selected aligned with clinical actions from observations of Stirling and MacLean [16] to understand the use of telehealth for these rehabilitation actions. Future studies should include additional questions aligned with the constructs of interest for their research goals.

## Conclusion

This survey of OTs and PTs during the early stages of the COVID-19 pandemic and revealed that the moderate increase in telehealth use as a care setting was accompanied by challenges and negative perceptions of telehealth tools that inhibited sustained use as a care setting for telerehabilitation. Participants were from a geographical sample that was primarily from the US but included international representation. Clinicians’ perceptions of and lack of access to telehealth tools contributed to limited adoption during the early pandemic. Respondents predominantly expressed great difficulty with their ability to perform a range of common cognitive, emotional, and physical assessment tasks via video-conferencing, and expressed the greatest difficulty with performing physical assessment tasks. These perceptions of difficulty may be due to various factors including the inability to capture robust visual or tactile information about body language or movements as well as limited viewpoints of a patient through video tools. Understanding the challenges perceived with individual tasks can help tailor technology capabilities to specific actions of the care process. Additional technology modalities (such as mobile and wearable technologies) and standardization of information collection and interpretation may provide more robust capabilities to augment telerehabilitation for remote patients and clinicians.

## Supporting information

S1 File. Full survey The full survey as administered is provided in this file. (DOCX)

S2 File. Dataset The full survey dataset used for analysis is provided in this file. (XLSX)
