# Supplementary material for: Telerehabilitation use and experiences in occupational and physical therapy through the early stages of the COVID-19 pandemic
Source: PLoS One. 2023 Nov 8;18(11):e0291605. doi: 10.1371/journal.pone.0291605 (PMC10631673; doi:10.1371/journal.pone.0291605)
Supplement: S1 File. Full survey — (DOCX) [file pone.0291605.s001.docx]

# **Supporting information**

**S1 File. Full Survey.** The full survey as administered is provided in this file.

Survey response types:

Circle indicates single-choice response.

Square indicates multiple-choice response allowed.

An open line indicates free response.

**Understanding Clinician Telemedicine Experiences During COVID-19**

Q00 **Usage of Telehealth for Occupational and Physical Therapists during the COVID-19 pandemic (HUM00182185)**
Principal Investigator: **Leia Stirling, PhD, Department of Industrial and Operations Engineering, Robotics Institute, University of Michigan**
You are invited to participate in a research study about the usage of telehealth prior to and during the COVID-19 pandemic. If you agree to be part of the research study, you will be asked to answer questions in this Qualtrics form. There are no direct Benefits of the research for you if you choose to participate. There are no direct Risks or discomforts for you if you choose to participate. There is no Compensation for participation.
Participating in this study is completely voluntary.  Even if you decide to participate now, you may change your mind and stop at any time. You may choose not to answer a survey question for any reason. Information collected in this project may be shared with other researchers, but we will not share any information that could identify you. If you have questions about this research study, please contact Prof. Leia Stirling (leias@umich.edu).
The University of Michigan Institutional Review Board Health Sciences and Behavioral Sciences has determined that this study is exempt from IRB oversight*.*

Start of Block: Biographical Info

Q1 What is your age?

- 18-34 (1)
- 35-64 (2)
- 65+ (3)

Q2 What is your sex?

- Female (1)
- Male (2)
- Non-binary (3)

Q3 What is your health care professional area?

- Occupational therapist (1)
- Physical therapist (2)
- Other (3) ________________________________________________

Q4 How long have you been practicing in healthcare?

- Less than 1 year (1)
- 1-5 years (2)
- 5-10 years (3)
- More than 10 years (4)

Q5 Please select the type(s) of cases you typically work with. [Select all that apply]

- Assistive Technology (1)
- Brain Injury or Neurology (2)
- Cardiovascular Rehabilitation (3)
- Driving and Community Mobility (4)
- Environmental Modification (5)
- Feeding, Eating, and Swallowing (6)
- Gerontology or Aging in place (7)
- Hand Therapy (8)
- Low Vision (9)
- Occupational Rehabilitation (10)
- Orthopaedics (11)
- Pediatrics (12)
- Physical Rehabilitation (13)
- Seated Mobility (14)
- Sensory Integration (15)
- Sports (16)
- Stroke Rehabilitation (17)
- Other (18) ________________________________________________

Q6 Please select the ages of patients you typically work with. [Select all that apply]

- Infant/Toddler (0-3 yrs) (1)
- Children (4-10 yrs) (2)
- Adolescents (11-18 yrs) (3)
- Adults (over 18 yrs) (4)
- Older Adults (over 65 yrs) (5)

Q7 Do you consider the area you are working in to be a rural, suburban, or urban community?

- Rural (1)
- Suburban (2)
- Urban (3)
- Other (4) ________________________________________________

End of Block: Biographical Info

Q8 Prior to COVID-19, in what care setting(s) did you support patients? [Select all that apply]

- Inpatient setting (1)
- Outpatient setting (2)
- In-home healthcare (3)
- Nursing home facility (4)
- School system (5)
- Telehealth (6)
- Other (7) ________________________________________________

Display This Question:

If Q8 = Telehealth

Q9 Prior to COVID-19, what reasons motivated you or your patient(s) to use telehealth? [Select all that apply]

- Distance to hospital (1)
- Transportation limitations (2)
- Reimbursement coverage availability (3)
- Concern for communicable diseases (4)
- Other (5) ________________________________________________

Q10 Prior to COVID-19, how many patients did you typically see in a week?

________________________________________________________________

Q11 Prior to COVID-19, how long would a typical clinical appointment be?

________________________________________________________________

Q12 Prior to COVID-19, how would you interact with patients? [Select all that apply]

- Face-to-face conversation (1)
- Direct interaction (e.g. physical cues) (2)
- Indirect interaction (e.g. virtual conversation, verbal instructions, etc.) (3)

Q13 Prior to COVID-19, how would you assess patient state or progress over the course of rehabilitation? [Select all that apply]

- Standardized tests (1)
- Visual observation of relevant tasks (2)
- Patient self-reports (3)
- Other (4) ________________________________________________

Q14 Prior to COVID-19, did you use any technology to assess patient motion (e.g. smartwatches, wearable sensors, camera-based motion capture, etc.)

- Yes (1)
- No (2)

Display This Question:

If Q14 = Yes

Q15 Prior to COVID-19, what technology did you use to assess patient motion?

________________________________________________________________

End of Block: Prior to COVID-19

Start of Block: Remote Work

Q16 During the COVID-19 pandemic, do you continue to spend face-to-face time with patients?

- Yes (1)
- No (2)

Display This Question:

If Q16 = Yes

Q17 Has your face-to-face case load changed due to COVID-19?

- I see more patients (1)
- I see about the same number of patients (2)
- I see fewer patients (3)

Display This Question:

If Q16 = Yes

Q18 Has your overall case load changed due to COVID-19?

- I see more patients (1)
- I see about the same number of patients (2)
- I see fewer patients (3)

End of Block: Remote Work

Start of Block: During COVID - All Subjects

Q19
During the COVID-19 pandemic, in what care setting(s) do you currently support patients? [Select all that apply]

- Inpatient setting (1)
- Outpatient setting (2)
- In-home healthcare (3)
- Nursing home facility (4)
- School system (5)
- Telehealth (6)
- Other (7) ________________________________________________

Q20 During the COVID-19 pandemic, how many patients do you typically see in a week?

________________________________________________________________

Display This Question:

If Q19 = Inpatient setting

And Q19 = Outpatient setting

And Q19 = In-home healthcare

And Q19 = Nursing home facility

And Q19 = School system

Q21 During the COVID-19 pandemic, how long would a typical clinical appointment currently be if conducted face-to-face (compared to face-to-face appointments before COVID-19)?

- Shorter now during COVID-19 pandemic (4)
- Same length during COVID-19 pandemic (5)
- Longer now during COVID-19 pandemic (6)

Q22 During the COVID-19 pandemic, have reimbursement policies changed for your patients or practice?

- Yes (1)
- No (2)

Display This Question:

If Q22 = Yes

Q23 What changes to reimbursement policies have you observed? [Select all that apply]

- More services covered (1)
- Services covered have increased reimbursement (2)
- Other (3) ________________________________________________

Q24 During the COVID-19 pandemic, what mode(s) of communication do you use to interact with patients? [Select all that apply]

- Face-to-face conversation/direct interaction (1)
- Phone Call (2)
- Video Call (3)
- Other (4) ________________________________________________

Q25 During the COVID-19 pandemic, how would you assess patient state or progress over the course of rehabilitation? [Select all that apply]

- Standardized tests (1)
- Visual observation of relevant tasks (2)
- Patient self-reports (3)
- Other (4) ________________________________________________

Q26 During the COVID-19 pandemic, have you used any technology to assess patient motion (e.g. smartwatches, wearable sensors, camera-based motion capture, etc.)

- Yes (1)
- No (2)

Display This Question:

If Q26 = Yes

Q33 During the COVID-19 pandemic, what technology have you used to assess patient motion?

________________________________________________________________

Q27 During the COVID-19 pandemic, have you used remote or virtual practice (telehealth, telemedicine, etc.) where you are not interacting with your patient in person?

- Yes (1)
- No (2)

Display This Question:

If Q27 = No

Q47 During the COVID-19 pandemic, what reason(s) motivated you to not use remote or virtual practice? [Select all that apply]

- Virtual communication technology is not available (1)
- Virtual communication technology is not effective or practical (2)
- Reimbursement policies don't cover remote/virtual practice (3)
- Other (4) ________________________________________________

Skip To: End of Survey If Q47 != Virtual communication technology is not effective or practical

Q48 If you were required to use video-conferencing, to what degree do you perceive being able to perform the following?

|  | Cannot perform (1) | Moderately difficult (2) | Slightly difficult (3) | Neither easy nor difficult (4) | Slightly easy (5) | Moderately easy (6) | Extremely easy (7) | N/A (8) |
| --- | --- | --- | --- | --- | --- | --- | --- | --- |
| Ask patients to perform specific activities to monitor their behavior (1) |  |  |  |  |  |  |  |  |
| Monitor patient fatigue (2) |  |  |  |  |  |  |  |  |
| Monitor patient frustration (3) |  |  |  |  |  |  |  |  |
| Monitor patient pain (4) |  |  |  |  |  |  |  |  |
| Provide verbal motion cues for my patients during tasks (5) |  |  |  |  |  |  |  |  |
| Assess patient active range of motion (6) |  |  |  |  |  |  |  |  |
| Assess patient manual dexterity (7) |  |  |  |  |  |  |  |  |
| Assess patient gross motor function (8) |  |  |  |  |  |  |  |  |
| Assess patient muscle tone (9) |  |  |  |  |  |  |  |  |
| Assess patient muscle activation patterns (10) |  |  |  |  |  |  |  |  |
| Assess balance (11) |  |  |  |  |  |  |  |  |
| Assess posture (12) |  |  |  |  |  |  |  |  |
| Assess ability to follow directions (13) |  |  |  |  |  |  |  |  |
| Assess compensatory mechanisms (14) |  |  |  |  |  |  |  |  |
| Assess coordination patterns within and across limbs (15) |  |  |  |  |  |  |  |  |
| Assess motion fluidity/smoothness (16) |  |  |  |  |  |  |  |  |
| Assess patient's ability to perceive objects and correctness of task performance (17) |  |  |  |  |  |  |  |  |
| Assess patient sensation (18) |  |  |  |  |  |  |  |  |
| Assess patient spasticity (19) |  |  |  |  |  |  |  |  |
| Assess patient strength (20) |  |  |  |  |  |  |  |  |

Skip To: End of Survey If Q48 [ N/A] (Count) >=

End of Block: During COVID - All Subjects

Start of Block: During COVID - Telemed

Display This Question:

If Q19 = Telehealth

Q28 During the COVID-19 pandemic, what reasons motivated you or your patient(s) to use telehealth? [Select all that apply]

- Distance to hospital (1)
- Transportation limitations (2)
- Reimbursement coverage availability (3)
- Concern for communicable diseases (4)
- Other (5) ________________________________________________

Display This Question:

If Q19 = Telehealth

Q29 During the COVID-19 pandemic, how long would a typical clinical appointment currently be if conducted via telehealth (compared to telehealth or face-to-face appointments before COVID-19)?

- Shorter now during COVID-19 pandemic (1)
- Same length during COVID-19 pandemic (2)
- Longer now during COVID-19 pandemic (3)

Display This Question:

If Q19 = Telehealth

Q30 During the COVID-19 pandemic, for telehealth appointments, have you seen the number of missed appointments change?

- Fewer missed appointments (1)
- Same (2)
- More missed appointments (3)

Display This Question:

If Q19 = Telehealth

Q31 Are the types of activities or exercises you prescribe for patients different for telehealth (versus face-to-face appointments)?

- Yes (1)
- No (2)

Display This Question:

If Q31 = Yes

Q32 Please describe the activities or exercises you would prescribe.

________________________________________________________________

Display This Question:

If Q24 = Phone Call

And Q24 = Video Call

Q34 If you are using phone or video calls to interact with patients, have you experienced any communications issues? [Select all that apply]

- Blurry video quality that makes it difficult to observe the patient (1)
- Patient and/or relevant body parts of patient are not fully visible in the camera view (2)
- The audio connection creates difficulty in understanding the patient's communications (3)
- Other (4) ________________________________________________

Display This Question:

If Q19 = Telehealth

Q35 Is there any new information you are gaining from virtual interactions compared to face-to-face interactions?

- Yes (1)
- No (2)

Display This Question:

If Q35 = Yes

Q36 Please describe what information you gain from virtual interactions compared to face-to-face interactions.

________________________________________________________________

Display This Question:

If Q19 = Telehealth

Q37 Is there any information you are missing from virtual interactions compared to face-to-face interactions?

- Yes (1)
- No (2)

Display This Question:

If Q37 = Yes

Q38 Please describe what information you are missing from virtual interactions compared to face-to-face interactions.

________________________________________________________________

Q39 When using video-conferencing, to what degree can you can perform the following?

|  | Cannot perform (1) | Moderately difficult (2) | Slightly difficult (3) | Neither easy nor difficult (4) | Slightly easy (5) | Moderately easy (6) | Extremely easy (7) | N/A (8) |
| --- | --- | --- | --- | --- | --- | --- | --- | --- |
| Ask patients to perform specific activities to monitor their behavior (1) |  |  |  |  |  |  |  |  |
| Monitor patient fatigue (2) |  |  |  |  |  |  |  |  |
| Monitor patient frustration (3) |  |  |  |  |  |  |  |  |
| Monitor patient pain (4) |  |  |  |  |  |  |  |  |
| Provide verbal motion cues for my patients during tasks (5) |  |  |  |  |  |  |  |  |
| Assess patient active range of motion (6) |  |  |  |  |  |  |  |  |
| Assess patient manual dexterity (7) |  |  |  |  |  |  |  |  |
| Assess patient gross motor function (8) |  |  |  |  |  |  |  |  |
| Assess patient muscle tone (9) |  |  |  |  |  |  |  |  |
| Assess patient muscle activation patterns (10) |  |  |  |  |  |  |  |  |
| Assess balance (11) |  |  |  |  |  |  |  |  |
| Assess posture (12) |  |  |  |  |  |  |  |  |
| Assess ability to follow directions (13) |  |  |  |  |  |  |  |  |
| Assess compensatory mechanisms (14) |  |  |  |  |  |  |  |  |
| Assess coordination patterns within and across limbs (15) |  |  |  |  |  |  |  |  |
| Assess motion fluidity/smoothness (16) |  |  |  |  |  |  |  |  |
| Assess patient's ability to perceive objects and correctness of task performance (17) |  |  |  |  |  |  |  |  |
| Assess patient sensation (18) |  |  |  |  |  |  |  |  |
| Assess patient spasticity (19) |  |  |  |  |  |  |  |  |
| Assess patient strength (20) |  |  |  |  |  |  |  |  |

Q40 For items that you cannot assess well with video-conferencing, what are challenges that you face?

________________________________________________________________

End of Block: During COVID - Telemed
